# The influence of seeding method and water depth on the morphology and biomass yield of farmed sugar kelp (*Saccharina latissima*) at a small-scale cultivation site in the northeast Atlantic

**DOI:** 10.1007/s10811-024-03394-9

**Published:** 2024-12-16

**Authors:** Catherine M. Wilding, Kathryn E. Smith, Carly L. Daniels, Jessica Knoop, Dan A. Smale

**Affiliations:** 1 School of Natural and Environmental Sciences, Newcastle University, Newcastle, NE1 7RU UK; 2 Marine Biological Association of the United Kingdom, The Laboratory, Citadel Hill, Plymouth, PL1 2PB UK; 3 College of Maths Science and Engineering, The University of Exeter, Penryn, Cornwall, TR10 9EZ UK; 4 Phycology Research Group, Department of Science, Ghent University, 9000 Ghent, Belgium

**Keywords:** Seaweed farming, Pheophyceae, Twine, Binder, Seeding technique, Vertical cultivation

## Abstract

Seaweed farming is a rapidly growing global industry, driven by increasing demand for biomass with a range of commercial applications. A major barrier limiting expansion of the industry is the need for cost-effective approaches to production. Established twine seeding methods are reliable, but recently developed binder methods offer potential scalability while minimising hatchery costs. Here, we compared growth of the farmed kelp *Saccharina latissima* using these two seeding methods. We also examined the influence of water depth on biomass production within a vertical cultivation system. Twine consistently achieved greater *S. latissima* biomass yield, with mean biomass almost four times higher than from binder seeding, and sporophytes reaching significantly greater density and total length. The biomass, length and density of *S. latissima* decreased with increasing water depth, a pattern which was more pronounced with twine seeding. We also observed morphological variability, with larger individuals on twine compared with binder treatments at all depths. Natural settlement of the non-target macroalga *Sacchoriza polyschides* was also recorded, at significantly greater biomass on binder treatments and at greater depths. Further work is needed to examine the predictability and extent of natural settlement of *S. polyschides*, and its potential as a commercially-viable species. Overall, twine seeding methods out-performed binder at a relatively dynamic, open coast, small-scale cultivation site. Moreover, while vertical cultivation systems can maximise yield relative to the spatial footprint of a seaweed farm, the marked reduction in cultivated biomass with increasing water depth should be considered within the local environmental context.

## Introduction

Global algal cultivation, predominantly seaweed, reached 35.1 million tonnes wet weight in 2020, accounting for almost 30% of total global aquaculture biomass (Araújo et al. 2021; Cai et al. 2021; FAO 2022). The sector is rapidly expanding in many regions (Kerrison et al. 2020) with emerging seaweed markets estimated to potentially be worth up to US$11.8 billion by 2030 (World Bank 2023). Europe is no exception, with expectations for substantial growth of the sector over the current decade (European Commission 2022, 2023). However, supply is dominated by imports from Asian cultivation (Araújo et al. 2021; Capuzzo 2022), with European production remaining heavily reliant on wild harvesting (Cai et al. 2021). As sustainable algal aquaculture can protect wild stocks from over-harvest and may provide a suite of ecosystem services (e.g., Kim et al. 2017; Hasselström et al. 2018) including habitat provisioning (Visch et al. 2020; Corrigan et al. 2022, 2024; Forbes et al. 2022), carbon capture (Duarte et al. 2022), nutrient cycling and eutrophication management (Farghali et al. 2023), there is broad interest in supporting expansion of the industry.

Kelp species (large brown seaweeds belonging to the order Laminariales) account for 47% of global seaweed production from cultivation (Cai et al. 2021) and have been the focus of research and development for the emerging European industry (e.g., Kerrison et al. 2015; Peteiro et al. 2016; Rolin et al. 2017; Bak 2019; Stanley et al. 2019). There are currently a number of farm layouts employed for kelp cultivation, including horizontal long lines or grid systems, and suspension of cultivation ropes vertically using dropper and head line systems (see reviews by Peteiro et al. 2016; Stanley et al. 2019; Wilding et al. 2021). The latter system is attractive because: (i) of its potential to produce relatively high biomass yield from a given spatial footprint; (ii) it can be readily adapted from existing infrastructure (e.g., mussel cultivation) with little need for modification; and (iii) it may be better suited to more wave-exposed environments (but see Gagnon 2024). However, the recognised impact of light attenuation with increasing water depth on seaweed growth (e.g., Smith et al. 2022; Boderskov et al. 2023) can limit production on vertical lines (Bak et al. 2018). Beyond site design, optimisation of all processes from hatchery to harvest, have received research interest with the aim of reducing production costs while maintaining or increasing yield, to facilitate commercialisation (e.g., Rolin et al. 2017; Bak et al. 2018; Kerrison et al. 2018a; Forbord et al. 2020; Boderskov et al. 2021, 2023; Corrigan et al. 2023).

The traditional method of seeding, known as “twining” involves spore seeding on to spools of twine followed by hatchery grow-out of kelp seedlings for around six weeks, when sporophyte lengths of ~ 10 mm are attained. Twine is then wrapped helically around cultivation ropes, either manually or mechanically (e.g., Solvang et al. 2021), for deployment at sea. This method is most efficient when using long cultivation ropes (e.g., horizontal long lines) rather than dropper systems, which require attachment of shorter lengths of twine to multiple vertical ropes (typically between 2 and 10 m in length). The recent development of a hydrocolloid binder (At-Sea Technologies, BE, now AtSeaNova) has the potential to minimise the hatchery and deployment costs associated with twine. Also referred to as direct seeding, the binder method combines microscopic gametophyte or embryonic sporophyte life stages with a binder suspension, which is then applied to cultivation substrates directly prior to deployment. This method provides the opportunity to use a wider variety of cultivation substrates such as nets and non-woven textiles and reduce hatchery grow-out time, which could save 13–23% of costs (Bak 2019) and once optimised use only 1% of the hatchery space required to for twine (Kerrison et al. 2018a). It also has the potential to help optimise stocking density (Kerrison et al. 2018a, 2020; Boderskov et al. 2021). The effectiveness of binder compared with traditional twine seeding has been tested in Denmark, Norway, Scotland and the Faroe Islands (Kerrison et al. 2018a, 2020; Bak 2019; Forbord et al. 2020; Boderskov et al. 2021) but, despite some promising results, yields attained from direct seeding are not consistently comparable to those attained from traditional twining. Further, the reliability of direct seeding with binder in dynamic coastal environments influenced by tidal currents and wave energy requires validation (Kerrison et al. 2018a, 2020; Forbord et al. 2020).

The sugar kelp *Saccharina latissima* is widely utilized for cultivation due to its fast growth rate, high productivity, and suitability for a wide range of applications (Kerrison et al. 2015; Peteiro et al. 2016; Bak et al. 2018; Araújo et al. 2021; Capuzzo 2022). While production of *S. latissima* has been increasing steadily along the North Atlantic coastline, there remains a need to optimise and/or automate seeding, deployment and harvesting techniques to improve cost-effectiveness. Moreover, within farm sites there is the need to better understand how small-scale environmental gradients such as depth and light availability affect biomass production, as has been observed in natural kelp populations (Smith et al. 2022). To address these knowledge gaps, we compared the success of *S. latissima* cultivation between twine and binder seeding methods, and across different water depths, at a mixed seaweed and mussel farm in Porthallow Bay (Cornwall, UK) over a typical growing season.

## Materials and methods

### Site characteristics

**Fig. 1:**
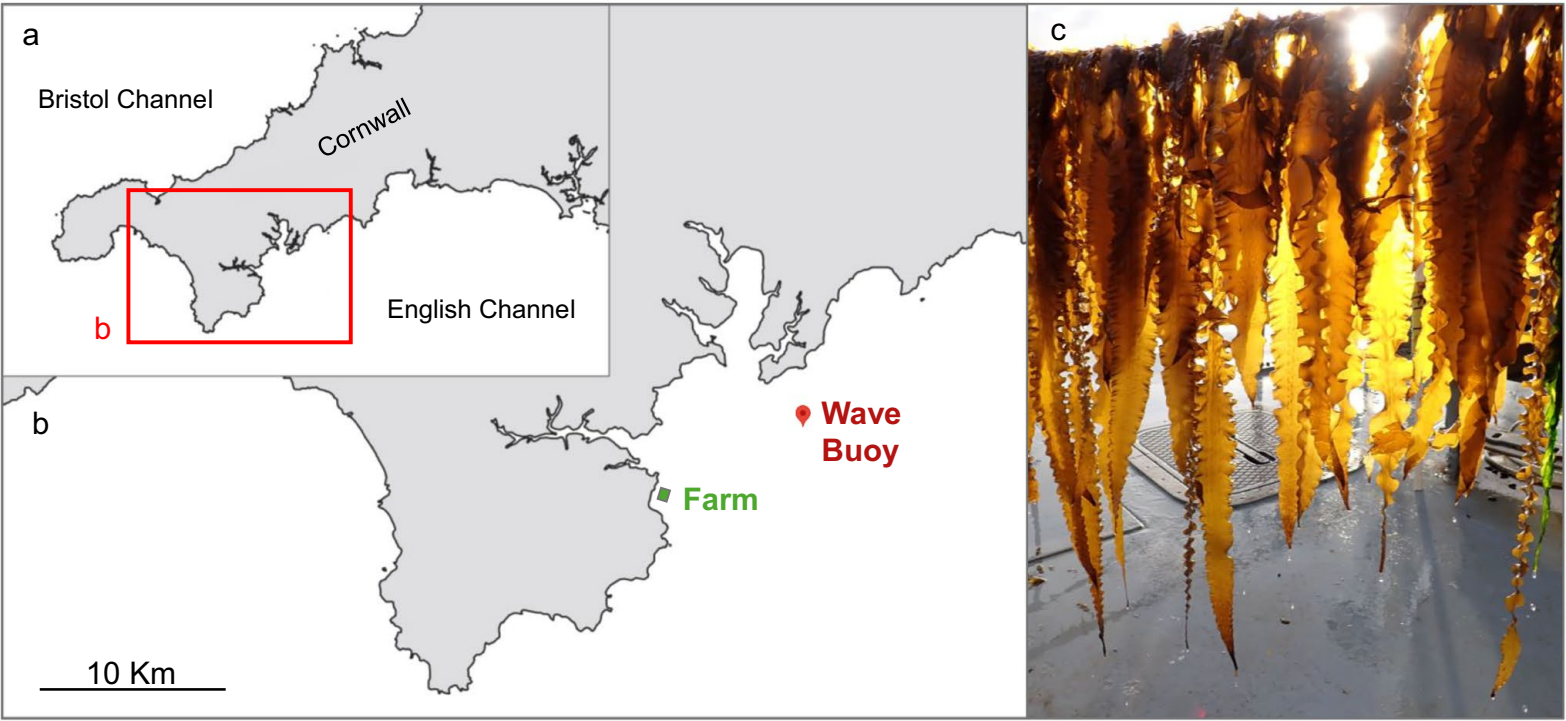
Study site map with: a) Location of the study area within the wider southwest England region, UK; b) Location of the Porthallow cultivation site relative to the FaBTest Wave buoy in Falmouth Bay; c) Representative *S. latissima* sporophytes cultivated at the farm from twine seeding treatments, captured in early spring (February)

Porthallow farm is a mixed seaweed and mussel farm located in Porthallow Bay, Cornwall, UK (50°04′36"N, 5°04′16"W; see Fig. 1). Farm infrastructure utilises a head line and dropper system, with 200 m horizontal longlines arranged in parallel to one another supporting vertical cultivation ropes. Head lines are suspended using tension anchors and large buoys at a constant depth of ~ 1 m below the sea surface. For further site details see Corrigan et al. (2023).

Environmental conditions were measured at the site throughout deployment. Temperature and light intensity data were collected using environmental sensors (HoBo pendant loggers, Onset, USA; data recorded every 30 min), with duplicate loggers deployed at 2 m and 5 m depth on an unseeded dropper rope attached to the same section of head line as the seeded ropes. Light was recorded in Lumens. Light sensors were cleaned during monitoring in February and March and only data for the 14 days following deployment or cleaning were analysed to remove any potential impacts from biofouling of the sensors (Smale et al. 2016). The site is sheltered from prevailing south-westerly winds and swell, but is exposed to significant sea states from the east and southeast resulting from wave fetch of ~ 300 km. Wave buoy data were retrieved from the FaBTest buoy in Falmouth Bay (~ 7.5 km northeast of the farm site, 50°06′24″N 4°58′49″W; see www.fabtest.com), which has a similar exposure to the site. The wave buoy was not operational between mid-January and early March, but all available data are presented.

### Seeding preparation

Four replicate dropper ropes for each of the twine and binder treatments were prepared for deployment on 26 October 2021. Each rope was 5.6 m long, allowing a 30 cm length to be left unseeded at each end for attachment to the farm infrastructure. In advance of seeding, ropes for both treatments were soaked in sterile seawater for 24 h to remove manufacturing residues and then air-dried for 72 h in a warm, well-ventilated space.

*Saccharina latissima* seed material was produced by Hortimare (B.V.) either as sporophytes growing on twine, or as a combined gametophyte and sporophyte suspension in culture media, both initially sourced from local populations. Nursery conditions are IP Hortimare (B.V.), where twine was seeded with gametophytes at 10 mg m^−1^, while for binder seeding density was 10 mg m^−1^ of gametophytes combined with 3,000 individual sporophytes m^−1^ following dilution with the binder suspension.

Twenty-four hours ahead of seeding, a 1% binder (AtSeaNova, B.E.) solution was prepared following manufacturer’s instructions. Gametophytes and sporophytes to be added to the binder solution were treated following standard protocols: flask lids were removed and contents agitated by gently swirling the flask by hand to re-suspend settled material and exchange the air in the flasks. Lids were then re-sealed, and containers stored on their sides in the dark at 4 °C overnight prior to seeding. Directly prior to seeding, the binder solution was diluted by addition of sterile sea water and the sporophyte and gametophyte solution, giving a 0.5% concentration binder-seaweed suspension. Twelve strand braided polyester cultivation ropes (14 mm diameter; Langman B.V.), were immersed into the suspension. The suspension was then massaged into the ropes and excess was removed by pulling the rope through a simple press roll device. Seeded droppers were stored out of direct sunlight in a lidded bucket for two hours before deployment at sea. Twine was supplied coiled around a PVC-tube spool, which was immersed into a 64 L tank of sterile seawater maintained at 10–11 °C with aeration overnight preceding seeding. Prior to seeding, ropes were soaked in sterile 11 °C seawater for one hour to avoid desiccation of seedlings. Manually, twine was wrapped helically around three strand twisted polyester cultivation ropes (14 mm diameter; Langman B.V.) and secured at both ends. A wrapping factor of 1.2 was used, so that the central 5 m of each dropper was seeded with 6 m of twine. Seeded droppers were sprayed with sterile seawater using a hand-pump spray to prevent desiccation and stored in a lidded bucket out of direct sunlight for two hours prior to deployment.

#### Deployment, monitoring and harvest

Seeded ropes were transported in separate sealed buckets for deployment (26 October 2021). Twine and binder dropper treatments were deployed alternately, spaced at 1.5 m intervals along a head line with each dropper positioned vertically in the water, weighted at the bottom with a brick (~ 1.7 kg). Following initial grow out, non-destructive length and density monitoring of *S. latissima* was conducted on 10 February and 14 March 2022. Farm access was not possible during April 2022. Monitoring was carried out in the top 0–1 m depth increment of each dropper. Within the uppermost 20 cm sub-section, the density of all adult sporophytes (defined as individuals > 15 cm in length) was recorded. Additionally, five representative large individuals were selected from within the meter and their total length recorded to give maximum attainable values.

On 5 May 2022, experimental droppers were harvested, transported ashore and allowed to drip-dry for ten minutes to remove excess water before processing. The total biomass of each dropper, and total biomass of each 1 m depth increment from the top of each dropper (i.e. 0–1 m, 1–2 m, etc.) were recorded. The density of adults was recorded within the first 20 cm sub-section of each depth increment and five large individuals were selected for morphometric measurements from the remaining meter, as described above. From these five, the length of the blade and stipe/holdfast complex were recorded separately for each individual. In addition to the target species, high rates of natural settlement of the large brown macroalga *Sacchoriza polyschides* were observed. Where found, data were recorded for this species as above for *S. latissima.* Adult *S. polyschides* individuals were defined as those with a holdfast engulfed by the bulb (Norton and Burrows 1969).

In addition to the main detailed study, *S. latissima* was also cultivated at the farm site in 2020 using binder seeding and again in 2023 using the twine method. Although not a fully crossed two factorial design, this allowed for a visual comparison of sporophyte density and total biomass from the 0–1 m depth increment at harvest across years/seeding methods. Methods from other years were standardised, with the exception of density where all sporophytes within each meter were counted in 2020, meaning that density estimates from 2022 and 2023 may be a slight overestimate by comparison. As temporal variation is to be expected, and this shallow depth band is susceptible to environmental variation (e.g., irradiance, waves), comparisons of different seeding methods from different years should be treated with caution, but are still indicative.

#### Statistical analysis

Two tailed t-tests were used to compare total dropper weight at harvest across seeding treatments for each species. For all other data collected at harvest, two-way fully crossed ANOVAs were carried out for each species independently, to explore the main effects of seeding treatment and depth, and their interaction term. Response variables for each depth increment were maximum blade, stipe/holdfast and total sporophyte length, adult density and wet weight biomass per meter of line. Across cultivation seasons the influence of seeding method on density and biomass are also presented, however formal statistics on these data were not feasible. All response variables were checked for homoscedasticity prior to analyses; data transformation was not needed. All statistical analyses and figures were produced in Rstudio v. 2022.12.0 (Rstudio Team 2020). All data are presented as mean values ± standard error (SE), and density values collected from 20 cm subsamples were standardised to per meter of line, to generate estimates that allow for comparison across years.

## Results

### Environmental conditions

**Fig. 2:**
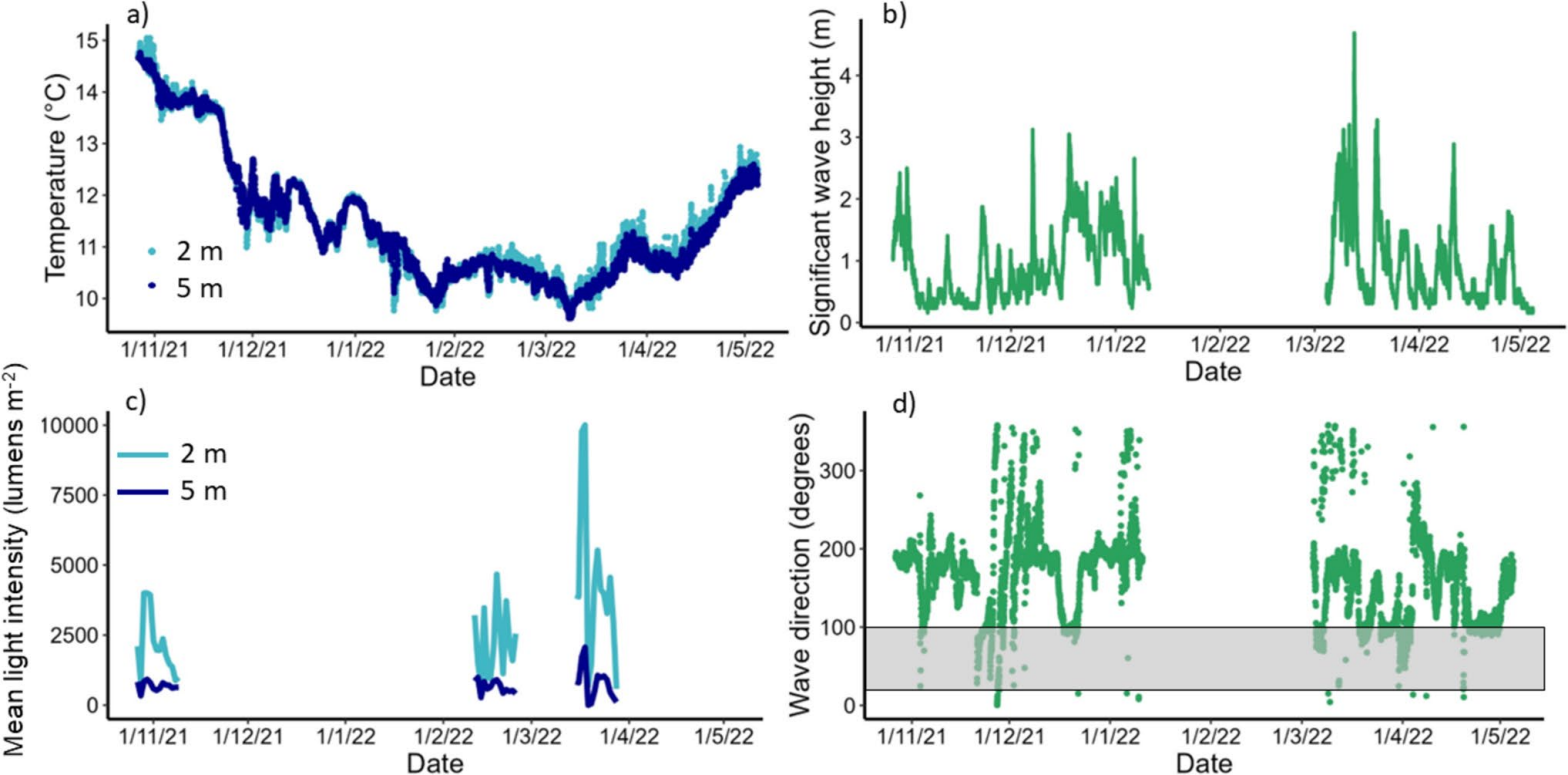
Environmental conditions recorded at the cultivation site, from seeded line deployment (late October 2021) through the cultivation season to harvest (early May 2022). a) Sea temperature at 2 m and 5 m water depth, b) wave height, c) mean light intensity at 2 m and 5 m water depth, and d) wave direction. Data in a) and c) were obtained from HoBo pendant loggers deployed at the farm site, whereas b) and d) are from the FaBTest Wave buoy in Falmouth Bay. The grey shaded area indicates waves directed towards the farm site within Porthallow Bay

Environmental variables followed a typical seasonal trend (Fig. 2). Sea water temperature declined from almost 15 °C at deployment in late October, to between 10–11 °C in February and March, before increasing through the spring until harvest time. Temperatures were comparable across depths, averaging 11.47 °C at 2 m and 11.45 °C at 5 m depth across the experimental period. Similarly, light levels were lower in autumn and early spring and increased through late spring until harvest. Light levels at 2 m were markedly higher and more variable than at 5 m, averaging 2874 lumens m^−2^ and 700 lumens m^−2^, respectively, across the observation period. Wave data showed frequent periods of increased significant wave height throughout the deployment period, including conditions of > 1 m swell directed towards the cultivation site within the first few weeks following deployment (Fig. 2). Significant wave heights exceeded 2 m on multiple occasions and exceeded 4 m in March 2022. However, Porthallow Bay was largely sheltered from the direction of the majority of wave activity, including periods of greatest wave heights (Fig. 2).

### Monitoring data

**Fig. 3:**
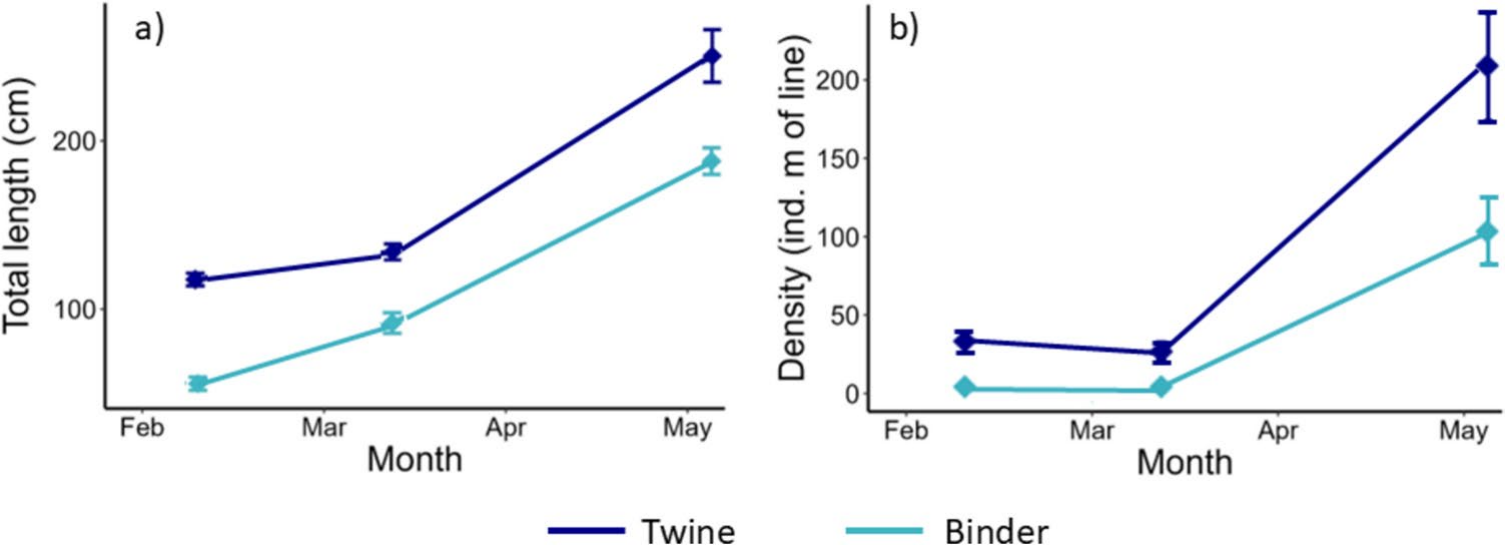
Time series monitoring data for *S. latissima* sporophytes cultivated at 0–1 m dropper depth for each treatment. Showing a) Mean (± SE) maximum total length of adult individuals (n = 5 sporophytes on each of 4 droppers), b) Mean (± SE) density of all adult sporophytes (> 15 cm total length) per meter of dropper line (n = 4 droppers)

In February 2022, following initial grow-out, the biggest adult *S. latissima* sporophytes within the 0–1 m depth increment were larger in the twine seeding treatments compared with the binder treatments (119.5 ± 3.7 cm and 55.8 ± 4.0 cm, respectively). Similarly, a higher density of adult *S. latissima* individuals was recorded in the twine treatments (30.0 ± 4.2 ind.m^−1^) compared with the binder treatments (1.3 ± 0.5 ind m^−1^), with the majority of sporophytes from binder being juveniles at this time. This trend was maintained across the monitoring period, with sporophytes in twine seeded treatments consistently achieving greater length and density values than binder seeded treatments (Fig. 3). Maximum sporophyte length increased at a broadly similar rate regardless of treatment over the growing season, while density increased markedly in twine treatments between the final monitoring point and harvest, presumably as sporophytes which were < 15 cm in March developed into adults by May.

### Harvest data

**Fig. 4:**
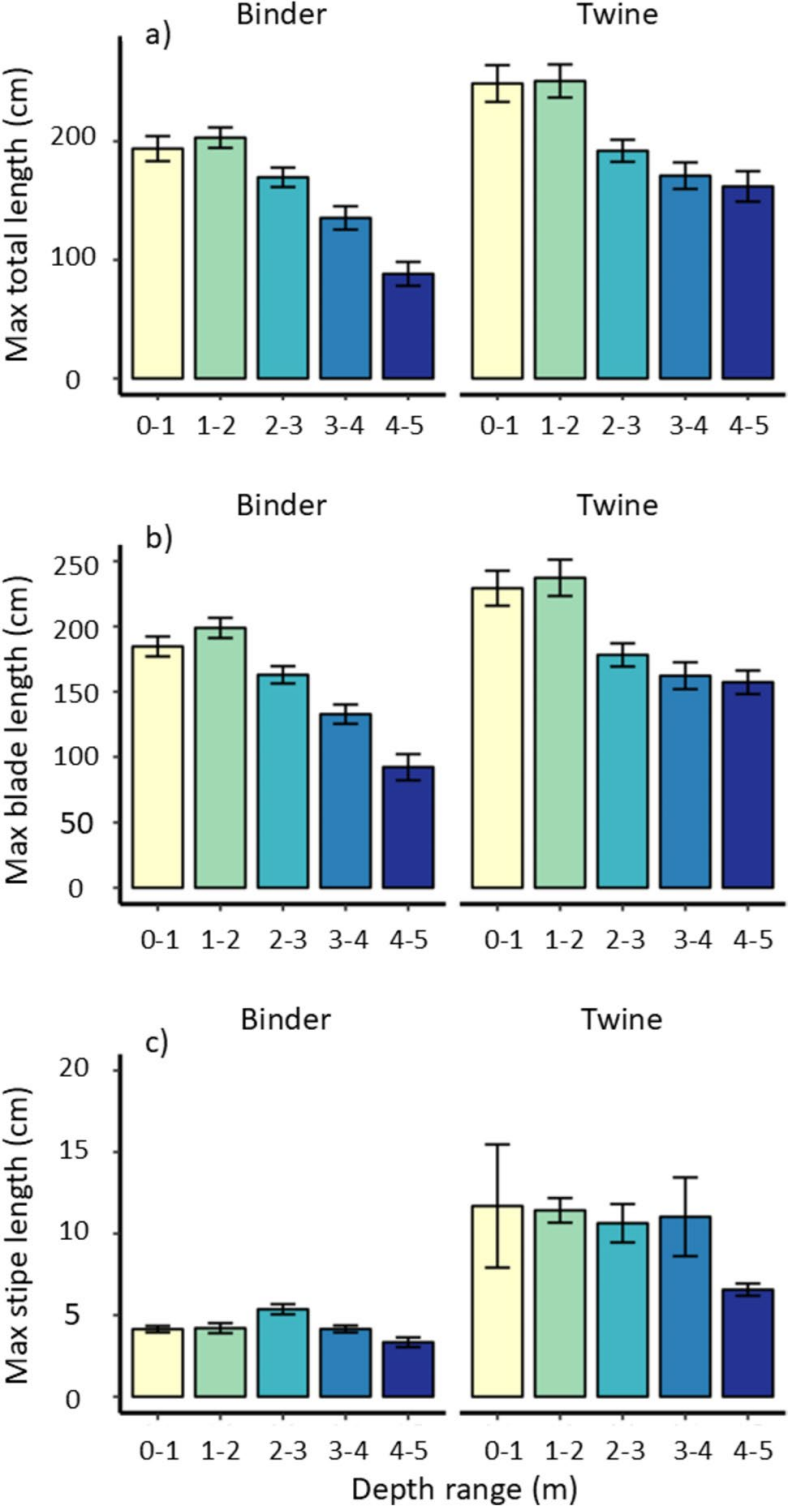
Mean (± SE) maximum length of cultivated *S. latissima* sporophytes at harvest (n = 5 individuals on each of 4 droppers), for each seeding treatment and depth increment. Showing a) total length, b) blade length, and c) combined stipe/holdfast length

At harvest, the maximum attainable total sporophyte length ranged from 250.8 ± 14.0 cm at 1–2 m depth on the twine treatment to 95.7 ± 9.8 cm at 4–5 m depth on the binder treatment, with marked variability between depths recorded for both treatments (Fig. 4). Similar patterns were observed for maximum blade length (Fig. 4). Statistically, maximum total length and blade length varied significantly across both treatments and depths (Table 1). Both metrics were higher on twine compared with binder treatments, and generally greatest in the shallowest two depth increments and lowest in the deepest two increments (Fig. 4). Maximum attainable stipe length varied significantly by treatment, depth, and the interaction of the two factors (Table 1). Stipes were longer in twine compared to binder treatments, and the effect of depth was more pronounced in the former (Fig. 4).

**Table 1:** Results of two-way ANOVAs to test for differences in cultivated *S. latissima* variables between seeding treatments and depth increments, and their interaction term. Also shown are response variables for the non-target species *S. polyschides.* Significant results (at P < 0.05) are shown in bold

	Treatment			Depth			Treatment*Depth			Residuals
	df	*F*	p	df	*F*	p	df	*F*	p	
*Saccharina latissima*										
Max. total length	1	48.10	** < 0.001**	4	28.88	** < 0.001**	4	1.38	0.242	149
Max. blade length	1	38.71	** < 0.001**	4	31.31	** < 0.001**	4	1.70	0.151	187
Max. stipe length	1	147.34	** < 0.001**	4	8.48	** < 0.001**	4	4.82	**0.001**	149
Density m^−1^	1	48.02	** < 0.001**	4	3.00	**0.034**	4	2.83	**0.042**	30
Wet weight m^−1^	1	94.50	** < 0.001**	4	16.01	** < 0.001**	4	8.99	** < 0.001**	30
*Saccorhiza polyschides*										
Density m^−1^	1	36.30	** < 0.001**	4	3.65	**0.012**	4	4.01	**0.009**	30
Wet weight m^−1^	1	22.40	** < 0.001**	4	2.08	0.096	4	1.88	0.140	30

**Fig. 5:**
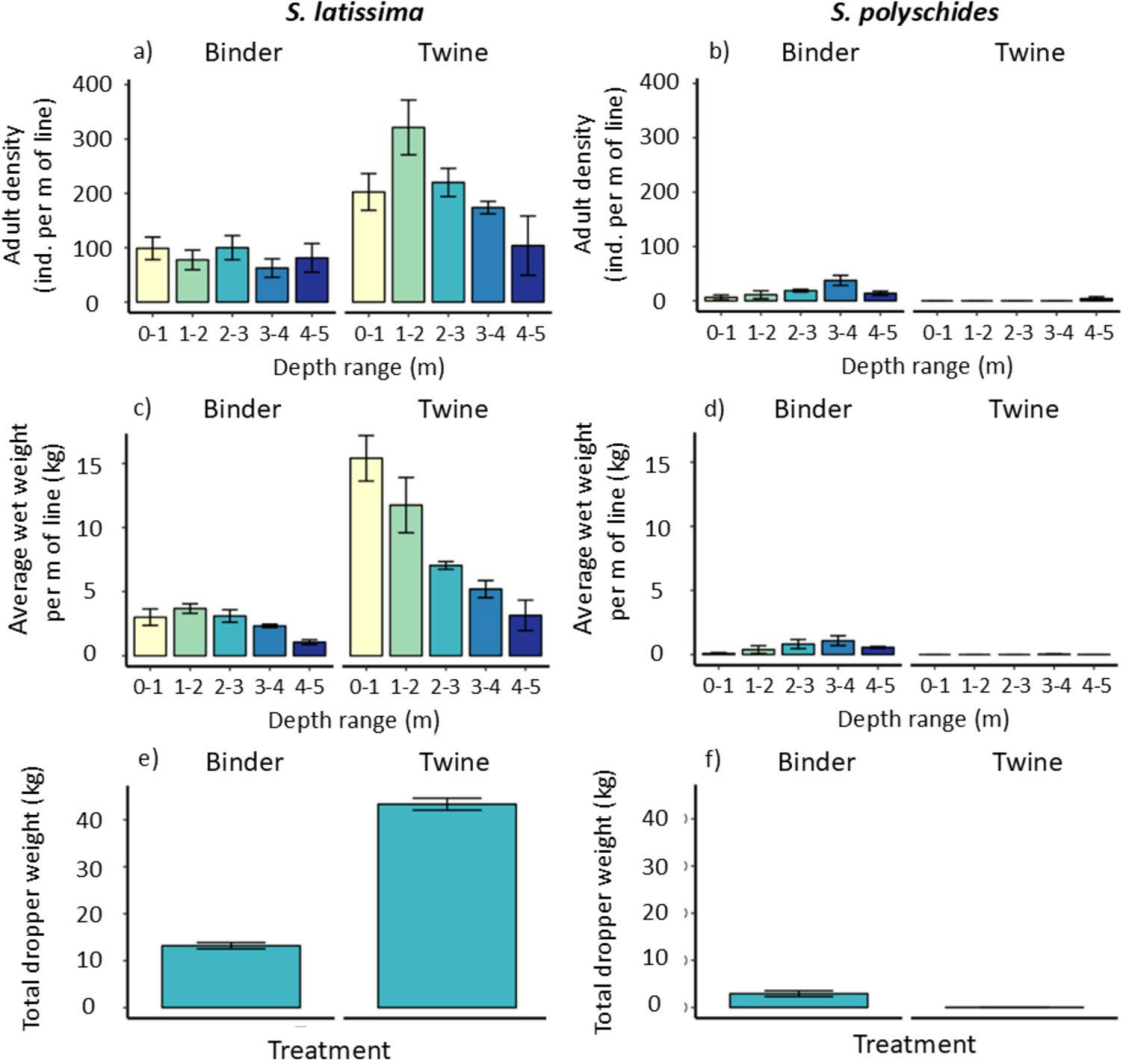
Mean (± SE, n = 4) a) & b) sporophyte density, c) & d) wet weight biomass, at harvest for each seeding treatment, depth increment and species (left = *S. latissima*; right = *S. polyschides*). e) & f) total biomass yield per dropper, for each seeding treatment and species

The density of adult *S. latissima* sporophytes at harvest ranged from a maximum of 64.3 ± 20.1 ind m^−1^ at 1–2 m depth on twine to a minimum of 12.5 ± 6.8 ind m^−1^ at 3–4 m depth on the binder treatment (Fig. 5). Statistically, density varied significantly with treatment, depth, and the interaction term (Table 1). The interaction was driven by a greater effect of depth on twine compared with binder, which exhibited relatively more consistency across depths (Fig. 5). For twine, density was greatest in the second depth increment (1–2 m) and then decreased continuously with increasing depth (Fig. 5). Importantly, the total wet weight biomass of *S. latissima* varied dramatically between seeding treatments, with biomass in the 0–1 m increment and 1–2 m increment being ~ 5 and ~ 3 times greater on twine compared with binder, respectively (Fig. 5). The main factors and their interaction term were significant (Table 1), with a stronger effect of depth observed for twine compared with binder seeding (Fig. 5). The total dropper wet weight biomass of *S. latissima* at harvest time was almost four times greater for twine than binder seeding treatments, with differences being highly significant (two tailed t-test: t(5) = 2.571, p < 0.001).

During the cultivation season, we observed high recruitment and growth rates of the non-target macroalga *S. polyschides*, a large brown seaweed that presumably originated from nearby source populations*.* At harvest, the density of *S. polyschides* sporophytes was markedly lower than that of the seeded *S. latissima*, reaching a maximum of 7.5 ± 3.7 ind m^−1^ at 3–4 m depth on the binder treatment (Fig. 5). The density of *S. polyschides* varied significantly between seeding treatment, depth, and the interaction term (Table 1), being significantly greater on binder compared with twine treatments, and the former exhibiting greater variability between depths (Fig. 5). In contrast with *S. latissima,* yield m^−1^ of *S. polyschides* was significantly higher on binder than twine treatments (Table 1), with a general trend of increasing wet weight biomass m^−1^ with increasing depth from binder treatments (Fig. 5). Similarly, total dropper wet weight biomass for *S. polyschides* was significantly higher from binder than twine seeding treatments (two tailed t-test: t(3) = 3.18, p = 0.018; Fig. 5).

**Fig. 6:**
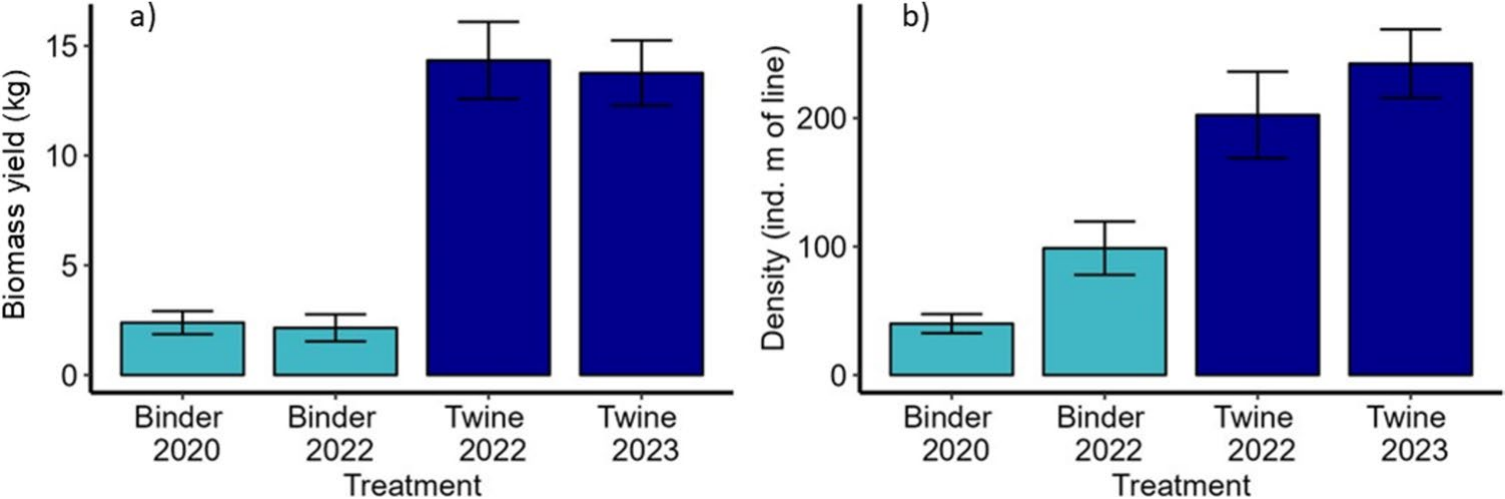
Mean (± SE; n = 4)* S. latissima* a) biomass yield, and b) sporophyte density, for the 0–1 m depth increment at harvest, for each seeding treatment across different cultivation years

When comparing across multiple cultivation seasons (within the shallowest 0–1 m depth increment only), both harvestable biomass and density of *S. latissima* was consistently greater on droppers that used twine rather than binder seeding methods (Fig. 6). On average, over the period of observation twine yielded ~ 5 times more biomass than binder seeding (Fig. 6).

## Discussion

Algal aquaculture is undergoing a period of rapid expansion globally, with focus on maximising efficiency and reducing costs to optimise production. We observed pronounced and significant differences in key metrics between seeding treatments. Twine consistently out-performed binder in terms of mean achievable *S. latissima* biomass, with a mean total dropper wet weight almost four times higher for twine than binder seeding at harvest. This trend was relatively consistent over time, both through the growing season (inferred from sporophyte size and density) and between years of observation. A combination of greater density and sporophyte length (particularly in the shallowest depth increments), and therefore harvestable biomass, underpinned the observed patterns. We also observed a marked reduction in biomass with increasing depth for *S. latissima* regardless of seeding method.

Across Europe, twine seeding is currently the principal method of kelp cultivation (Kerrison et al. 2018a, 2019b; Stanley et al. 2019; Forbord et al. 2020). It is reliable because at deployment seedlings are already firmly attached, likely to be of larger size, and are correctly orientated (Forbord et al. 2020). By contrast, binder methods are still in their infancy. Despite potential cost and timesaving benefits, binder seeding is presently unreliable and has been described as ‘hit or miss’ even by researchers who have demonstrated its success in certain contexts (e.g., Kerrison et al. 2018a). Compared with twine seeding, binder seeding may result in many seedlings being poorly orientated (Kerrison et al. 2018a), exhibiting retarded growth (Umanzor et al. 2020) or delayed blade growth because of the early energy investment in holdfast development (Forbord et al. 2020; Boderskov et al. 2021). Additionally, although the viscosity of binder is designed to hold seedlings in place while they form an attachment, poor performance of binder compared with twine may result from greater vulnerability to current flow or waves, which can dislodge developing sporophytes before they have attached securely, resulting in low yields (Mols-Mortensen et al. 2017; Kerrison et al. 2020). The minimum time required for successful attachment before the binder is washed away has not been empirically examined but a week-long period of calm weather following deployment is recommended in the literature (Kerrison et al. 2020). Improving seeding success by anticipating calm weather following deployment remains a key challenge for seaweed farmers. This constraint significantly limits the potential advantages of binder technology, and farmers require seeding systems that offer reliability under a broader range of conditions.

Relative to most other sites where the two seeding methods have been compared, where fetch and wave exposure were limited (e.g., Kerrison et al. 2018a, 2020; Forbord et al. 2020; Boderskov et al. 2021), our study site in Porthallow Bay is moderately wave-exposed. Although sheltered from the most severe waves (which exceeded 4 m significant wave height in nearby Falmouth Bay), resulting swells still wrap into Porthallow Bay to some extent. Indeed, the farm was directly exposed to multiple periods where significant wave height exceeded 1 m during the first few weeks following deployment, which may explain the reduced density of sporophytes observed from binder seeding and from within the shallowest depth increment from twine seeding. Wave surge may have had a disproportionate effect on binder, resulting in the poor performance of this treatment compared with other studies (e.g., Kerrison et al. 2018a, 2020; Boderskov et al. 2021). It may be, therefore, that a calm weather window at deployment is more critical to the success of binder seeding at moderately wave-exposed sites compared with more wave-sheltered locations.

The comparatively poor results achieved with binder in the present study relative to others in the literature may also be due to cultivation substrate. A benefit of binder is that it can be used on substrates which are not suitable as carriers for twine, such as nets and non-woven textiles, provided they are sufficiently absorbent. Studies reporting high yields from binder seeding have used ribbon substrates (Kerrison et al. 2018a, 2020; Bak 2019) which provide a larger surface area (e.g., a 50 mm width compared to 12 mm or 18 mm diameter) than rope and have been specially developed to enhance sporophyte bio-adhesion (see AlgaeRibbon, IP copyright AtSeaNova BV). By contrast, although specially designed seaweed cultivation ropes do exist (e.g., AlgaeRope IP copyright AtSeaNova BV), for twine seeding the use of widely available polypropylene rope is common, and polypropylene ropes have a smooth texture which is likely a poor surface for holdfast bioadhesion (Kerrison et al. 2018b, 2019a; 2020). Similar sporophyte retention for binder seeded AlgaeRope and twine seeded polypropylene has previously been reported, with 1–1.5% of the seeded sporophytes retained on each (Kerrison et al. 2020). This improved to about 5% with AlgaeRibbon, and these differences were reflected in the subsequent *S. latissima* yields. It may be therefore that the texture and width of the substrate are important drivers of the high binder yields reported elsewhere. Our results are also similar to those of Forbord et al. (2020), in that rope structure may have confounded the difference between twine and binder treatments. In both studies twine had a twisted structure and was wound around twisted rope substrate for deployment at sea, whereas binder was applied onto braided ropes (both 14 mm diameter polyester), which could have impacted the final yield (Forbord et al. 2020). Twisted twine structures have been found to perform significantly better than braided ones during hatchery cultivation (Kerrison et al. 2019b), so the same may extend to rope structure at sea.

In addition to seeding methods (e.g., Kerrison et al. 2018a, 2020; Bak 2019; Forbord et al. 2020; Boderskov et al. 2021), various other factors have also previously been identified as influencing cultivation success. These include environmental conditions (Peteiro and Freire 2013; Kerrison et al. 2015; Mols-Mortensen et al. 2017; Boderskov et al. 2021), deployment time (Boderskov et al. 2021, 2023; Nardelli et al. 2024), developmental stage at deployment (Kerrison et al. 2018a; Forbord et al. 2020) and the cultivation substrate used (Kerrison et al. 2018a, 2019b; 2020; Bak 2019; Boderskov et al. 2023). For example, regardless of seeding method, calm conditions are more likely to contribute to success than rough conditions during the early period following deployment (Mols-Mortensen et al. 2017; Forbord et al. 2020), while increased water movement during hatchery grow-out can prime developing sporophytes, improving growth and yield at wave or current exposed farm sites (Buck and Grote 2018; Nardelli et al. 2024).

Natural kelp populations in the study region of southwest UK generally persist no deeper than 10 m below chart datum (Smith et al. 2022), and maximum cultivation depths of 5–7 m are recommended for kelp species in UK waters (Kerrison et al. 2015). As such, the dropper depths used in the present study are close to this maximum operational depth limit, with a clear reduction in light intensity between 2 and 5 m water depth (Fig. 2). The pronounced trend of decreasing biomass with increasing depth for twine seeded *S. latissima* in our study is consistent with findings from natural kelp populations (e.g., Smith et al. 2022), and those from cultivation on both horizontal longlines (Boderskov et al. 2023) and vertical systems (Bak et al. 2018). Boderskov et al. (2023) found that horizontal lines grown at 3.5 m depth produced a biomass yield only 26% of that achieved from 1.5 m depth in Denmark. In vertical cultivation systems in the Faroes, where water clarity is higher, biomass yield at 3.5 m only decreased to 88% of that from 1.5 m, and cultivation may be possible to depths of 19 m (Bak et al. 2018). The presence of dense sporophytes growing on the first few meters of rope in vertical cultivation systems is likely to exacerbate light limitation on the individuals growing directly beneath through shading, in addition to natural light attenuation through the water column. Shading by cultivated *S. latissima* likely increased through the cultivation season as individual sporophytes increase in size. This shading effect has been reported to be as much as 40% of surface irradiance at 5 m depth, at the peak of the seaweed biomass (i.e. just before harvest) in Sweden (Visch et al. 2020). Seasonal variation in the effect of depth on cultivated seaweed growth and biomass production has also been reported (Bak et al. 2018). While vertical cultivation systems clearly have the potential to be more productive per unit area of seabed than horizontal systems, taken together these findings emphasise the need for prospective farmers to consider the impact of depth and local water clarity when estimating yield and choosing the most appropriate system design. Intended use of the biomass should also be considered, as cultivation rope orientation (i.e. vertical versus horizontal) has also been found to impact sporophyte morphology (Peteiro and Freire 2011) and will likely influence the structure of fouling communities (Corrigan et al. 2024).

Sporophyte density can affect growth, morphology and total achievable yield (Forbord et al. 2020 and references therein; Kerrison et al. 2020). In *S. latissima,* morphology and weight are strongly correlated with density, with longer stipe lengths being reported at high densities, due to resource limitation and competition for light (Kerrison et al. 2020). In the present study, twine seeded *S. latissima* achieved maximum lengths at 0–1 m depth, while the maximum density was observed at 1–2 m depth. This indicates that at 0–1 m the biomass is comprised of fewer, larger specimens, while at 1–2 m biomass consists of shorter individuals in greater abundance. These findings could be the result of higher dislodgement due to wave exposure from 0–1 m than 1–2 m, the negative impacts of high U.V. radiation, or mechanical shear from floating debris at the shallowest depth. For binder seeded *S. latissima* the opposite pattern was observed, with the longest individuals being recorded below 1 m depth, no clear trend in density, and densities substantially lower than those from twine seeded treatments. At comparable depths, *S. latissima* stipe lengths were consistently greater on twine seeded treatments than on binder, which broadly corresponds to the higher densities recorded from twine treatments and is consistent with findings from elsewhere (e.g., Kerrison et al. 2020). Morphological responses can be important to seaweed farmers, depending on the application of the cultivated biomass, with specific characteristics (size or texture) sometimes desirable for food uses (e.g., Peteiro and Freire 2011, 2013), while attaining maximum biomass yield is prioritised by most applications, such as for biofuel production (discussed in Kerrison et al. 2020). Availability of biochemicals is also likely to be affected by changes to morphology if, for example, a target compound is produced in a particular part of the sporophyte. The optimal cultivation density for achieving maximum yield is not yet known for any kelp species (Kerrison et al. 2015) and will depend on the local environmental context and the intended end-use of the cultivated biomass. Clearly, further work is needed to develop seeding and grow-out techniques to optimise stocking density.

Seeding method had a significant effect on the yield of *S. polyschides,* which exhibited higher density and biomass values on binder treatments compared with twine, and at greater depths. As an opportunistic coloniser, the reduced density of *S. latissima* from binder treatments and at greater depths is likely to have facilitated increased *S. polyschides* settlement and growth due to lower competitive pressure from *S. latissima*. Light attenuation is also likely to be a key driver of the responses observed with depth (Fig. 2), as *S. polyschides* may have lower light requirements and be better adapted to growth at greater depths compared with *S. latissima* (Kerrison et al. 2015; Smith et al. 2022). Another possible explanation for increased density on binder lines could be the presence of the binder acting as a settlement cue. Further research could compare natural settlement from untreated ropes with those soaked with binder but not seeded.

Currently, European kelp cultivation is most established for *S. latissima* (Kerrison et al. 2015; Peteiro et al. 2016; Forbord et al. 2018; Bak 2019; Araújo et al. 2021), and remains underdeveloped for *S. polyschides*, despite its extremely fast growth rate and suitability for various applications. There is an increasing need for species diversification in seaweed farming and in aquaculture more broadly, to upscale and meet increasing demand (Grebe et al. 2019; Goecke et al. 2020; Huntington and Cappell 2020; Kim et al. 2022). Species which settle naturally from wild populations may represent an opportunity to reduce costs by eliminating the hatchery phase, although further investigation into variation in natural settlement processes would be needed to allow for reliable exploitation by cultivators. *S polyschides* is a warm-adapted species which has been reported to have recently increased in abundance in the UK (Salland et al. 2023) and may have undergone poleward range expansions (Birchenough and Bremner 2010). As such, developing cultivation of this macroalga could boost climate resilience strategy in the emerging seaweed cultivation sector. The size of individuals at harvest time from the present study (maximum biomass of 684 g) is comparable with those from wild populations from the region (e.g., Salland et al. 2023). Although seasonal, this species can attain much greater biomass and lengths of 210 cm (Norton and Burrows 1969), exhibiting great cultivation potential. *S polyschides* is edible (e.g., Lodeiro et al. 2012; Cardoso et al. 2023) and a known source of enzymes (Almeida et al. 1998), anti-inflammatory molecules (Cardoso et al. 2023), fatty acids (Barbosa et al. 2020), bioactives for cosmetics (Susano et al. 2022), minerals (Rey-Crespo et al. 2014), alginate (Silva et al. 2015; Kaidi et al. 2022), used in nano-particle production (González-Ballesteros et al. 2021), and as a fertilizer (Soares et al. 2020). However, the species is not listed as a food under either the UK or EU regulation of novel foods (2015/2283) (EU 2015), which is a prerequisite for many markets. If feasible, (i.e. for a milled, mixed kelp species product which would not require labour intensive separation of species at harvest time), and subject to inclusion in regulation which would permit sale, we advocate that natural settlement of *S. polyschides* could be encouraged and utilised, rather than considered problematic.

In conclusion, binder seeding methods clearly require further development and validation, although the potential cost and time savings of the method may still be realised, particularly as seaweed cultivation progresses towards larger scales. The initial growth advantage of twine may be less evident under different environmental conditions (e.g., more wave sheltered than Porthallow), or with improvements to pre-deployment culturing. In lieu of improvements to reliability of binder methods, the use of traditional twine is necessary for now to achieve reliable yields and allow the seaweed industry to continue to develop in the UK and Europe. Furthermore, while vertical cultivation systems have the potential to be more productive per unit area of seabed than horizontal systems, our findings provide empirical evidence of the reduction in biomass with depth in vertical systems. As strong drivers of seaweed productivity, the impact of depth and local water clarity must be considered by prospective farmers in forecasting expected yield and choosing the most appropriate system for any given location. Combining twine seeding with vertical site layouts which use continuous cultivation lines in a “castellation” or “garland type” (Peteiro et al. 2016) system, or optimising seeding methods for vertical net substrates (Boderskov et al. 2023) has the potential to maximise yield and efficiency. Finally, further investigation into the predictability and utility of opportunistically-settling species such as *S. polyschides* could contribute to the expansion of the seaweed industry.

## Data Availability

Access to data and code is available on request from the lead author.
